# The Lasso Segment Is Required for Functional Dimerization of the *Plasmodium* Formin 1 FH2 Domain

**DOI:** 10.1371/journal.pone.0033586

**Published:** 2012-03-13

**Authors:** Alexander Ignatev, Saligram Prabhakar Bhargav, Juha Vahokoski, Petri Kursula, Inari Kursula

**Affiliations:** 1 Department of Biochemistry, University of Oulu, Oulu, Finland; 2 Biocenter Oulu, University of Oulu, Oulu, Finland; 3 Department of Chemistry, Centre for Structural Systems Biology, Helmholtz Centre for Infection Research, University of Hamburg, and German Electron Synchrotron (DESY), Hamburg, Germany; University of Heidelberg Medical School, Germany

## Abstract

Apicomplexan parasites, such as the malaria-causing Plasmodium species, utilize a unique way of locomotion and host cell invasion. This substrate-dependent gliding motility requires rapid cycling of actin between the monomeric state and very short, unbranched filaments. Despite the crucial role of actin polymerization for the survival of the malaria parasite, the majority of *Plasmodium* cellular actin is present in the monomeric form. *Plasmodium* lacks most of the canonical actin nucleators, and formins are essentially the only candidates for this function in all *Apicomplexa*. The malaria parasite has two formins, containing conserved formin homology (FH) 2 and rudimentary FH1 domains. Here, we show that *Plasmodium falciparum* formin 1 associates with and nucleates both mammalian and *Plasmodium* actin filaments. Although *Plasmodium* profilin alone sequesters actin monomers, thus inhibiting polymerization, its monomer-sequestering activity does not compete with the nucleating activity of formin 1 at an equimolar profilin-actin ratio. We have determined solution structures of *P. falciparum* formin 1 FH2 domain both in the presence and absence of the lasso segment and the FH1 domain, and show that the lasso is required for the assembly of functional dimers.

## Introduction

Apicomplexan parasites comprise an important group of human and animal pathogens. Best-characterized members of this *phylum* are the causative agents of malaria (*Plasmodium spp.*) and toxoplasmosis (*Toxoplasma gondii*). These pathogens share a unique mode of actin-dependent motility, characterized by the absence of any specialized organelles or obvious changes in the cell shape [1]. Instead, this so-called gliding motility involves peculiarly short, unstable actin filaments, which work in concert with an unconventional myosin, and a small set of regulatory proteins, governing the rapid cycling of actin monomers back to the growing end of the filament [2]. This miniature machinery lies in a confined space between the plasma membrane and the inner membrane complex of the parasite [3], [4] and is linked, through the plasma membrane, to as-of-yet unidentified host cell receptors and, through the inner membrane complex, to microtubules in the cytoplasmic compartment of the parasite [3].

Despite the importance of actin polymerization for the motility and invasion – and thus survival – of these parasites, most of their cellular actin is present in monomeric form, and short actin filaments are formed only transiently [5]. Actin polymerization in *Apicomplexa* is controlled by a surprisingly small set of regulatory proteins and, for example, most of the conventional actin filament nucleators, such as the Arp2/3 complex and Spire proteins are not present. The core actin regulators in *Plasmodium* include two formins [6], one profilin [7], two actin depolymerization factors [8], [9], two capping protein subunits [10], coronin [11], and a C-terminal cyclase-associated protein homologue [12].

Formins are a family of large multidomain proteins, important in many biological processes involving actin polymerization and, as recently discovered, also microtubule dynamics (reviewed in [13]–[15]). They are characterized by the presence of two formin homology (FH) domains: the filamentous (F)-actin-binding FH2 domain [16], [17] and the proline-rich FH1 domain, which in turn interacts with actin monomers indirectly *via* other regulatory proteins, such as profilins [18]–[20]. Formins work, on the one hand, as actin nucleators and, on the other, as processive cappers, which, together with profilins, also accelerate filament growth *via* recruiting a large pool of monomeric actin close to the barbed (growing) end of the filament [16], [21], [22]. Previous studies, mainly on mammalian and yeast formins belonging to the mDia family, have shown that the core FH2 domain alone inhibits actin polymerization, most likely *via* binding to the barbed end and preventing addition of new actin monomers rather than *via* monomer sequestering [21], [23]. The inclusion of an additional region N terminal of the core FH2 domain dramatically changes the properties of formins, turning them into efficient polymerization catalysts [23]. This so-called lasso region, linking the FH1 and FH2 domains, has been shown to form almost the sole contact upon the formation of a flexible dimer of FH2 domains [24]. Although the dimeric lasso-containing FH2 domain is sufficient for both nucleation and elongation of actin filaments [16], [21], for most formins, profilin has been shown to act as a further accelerator, speeding up polymerization by as much as nearly 20-fold [19]. Formins differ greatly in the number of proline-rich repeats in their FH1 domains and, thus, in the way they recruit profilin-actin. Different formins also utilize different profilin isoforms [25].

Apicomplexan genomes encode two to three FH2-domain-containing formins and a single profilin isoform. *Plasmodium falciparum* profilin (*Pf*-Pfn) binds to proline-rich sequences [7], indicating that the profilin-formin-mediated regulation of actin polymerization may be conserved. However, the two *Plasmodium* formin isoforms contain only rudimentary FH1 domains, and it is not clear whether they really participate in the regulation of actin dynamics in a profilin-dependent manner. In addition, *Plasmodium* has a nuclear formin-like protein, which also has a conserved FH2 but no FH1 domain [26]. What is more, none of the regulatory domains conserved in animal and yeast formins are present in the apicomplexan counterparts.

*P. falciparum* formin 1 (*Pf*-Frm1) and *T. gondii* formins induce actin filament formation *in vitro* using mammalian actin [6], [27]. However, until now, it has not been known if the parasite formins display the same activity also towards parasite actins, which have dramatically different polymerization properties. Here, we show that *Pf*-Frm1 nucleates actin filaments and works on both mammalian skeletal muscle and *Plasmodium* actins *in vitro*. *Pf*-Pfn alone sequesters actin monomers, inhibiting polymerization, and has little or no effect on polymerization in the presence of different *Pf*-Frm1 variants. *Pf*-Frm1 dimerizes in solution, and the lasso domain, preceding the core FH2 domain, is required for the assembly of functional dimers, and thus, the nucleation activity of *Pf*-Frm1.

## Materials and Methods

### Cloning and recombinant protein expression and purification

The gene encoding *P. falciparum* formin 1 FH1 and FH2 domains (*Pf*-Frm1-FH1FH2; (amino acids 2203–2675)) was amplified from genomic DNA 3D7 clone of *P. falciparum* and inserted into the pETM-11 (EMBL) vector using the *Nco*I and *Xho*I restriction sites. Two shorter constructs, lacking the FH1 domain or the FH1 domain and the so-called lasso segment (*Pf*-Frm1-FH2; amino acids 2242–2672 and *Pf*-Frm1-FH2Δlasso; amino acids 2272–2672, respectively) were amplified from this vector, and inserts were assembled using multi-tier sequence and ligation independent cloning (SLIC; [28]) into a PCR-linearized pETM-14 (EMBL) vector followed by annealing. The plasmids were transformed into *Escherichia coli* Rosetta(DE3) (Novagen), the recombinant proteins were expressed using 1 mM isopropyl-β-*D*-1-thiogalactopyranoside-induction at 18°C overnight in lysogeny broth (LB). The hexa-histidine (6×His)-tagged formins were purified on a Ni-NTA column (GE Healthcare) under native conditions in a buffer containing 50 mM sodium phosphate (pH 7.5), 300 mM NaCl, and 5 mM β-mercaptoethanol. A gradient of 10 to 500 mM imidazole in the same buffer was used for eluting the proteins. The 6×His-tags were cleaved during overnight dialysis against 40 mM tris(hydroxymethyl)aminomethane (Tris)-HCl (pH 7.5), 100 mM NaCl, 10% glycerol, and 1 mM dithiothreitol (DTT) in the presence of either tobacco etch virus (*Pf*-Frm1-FH1FH2) or human rhinovirus 3C (*Pf*-Frm1-FH2 and *Pf*-Frm1-FH2Δlasso) protease. Uncleaved proteins and 6×His-tagged proteases were removed by binding to a Ni-NTA column, and the flow-through fractions were concentrated and applied to a Superdex 200 16/60 column (GE Healthcare), equilibrated with 40 mM Tris-HCl (pH 7.5), 100 mM NaCl, 10% glycerol, and 1 mM DTT. Purified proteins were concentrated and stored at −70°C until use in assays.

The gene encoding *Pf*-Pfn was cloned into the pGAT-2 vector [29], which contains a thrombin cleavage site after the 6×His-glutathione-*S*-transferase (GST) tag, using the *Nco*I and *Xho*I restriction sites. The protein was expressed in *E. coli* BL21(DE3), and the 6×His-GST-*Pf*-Pfn was purified essentially as described before [7], using a HisTrap column (GE Healthcare). After dialysis into 10 mM Tris-HCl (pH 7.5), 100 mM NaCl, and 5% glycerol, *Pf*-Pfn was released from the 6×His-GST tag using thrombin (GE Healthcare). The cleaved *Pf*-Pfn was then passed through a HisTrap column, and the flow-through was subjected to size exclusion chromatography (SEC) over a Superdex 200 16/60 column (GE Healthcare) in 10 mM Tris-HCl (pH 7.5) and 100 mM NaCl. Peak fractions were collected, concentrated, and stored on ice for subsequent experiments.

Genes encoding *P. falciparum* actin 1 (*Pf*-Act1) and *P. berghei* actin 2 (*Pb*-Act2) were synthesized and codon-optimized for insect cell expression by Mr. Gene (now part of Invitrogen). The *Pf-Act1* gene was cloned into the pFastBac-HTA vector (Invitrogen) using the *Nco*I and *Xho*I sites. The cloned plasmid was transformed into DH10Bac (Invitrogen) cells for bacmid generation. Sf9 (Invitrogen) cells were transfected with the *Pf-Act1* bacmid using the FuGene 6 transfection reagent (Roche), and virus particles were harvested after 3 days of infection and used to generate a high-titer viral stock. Typically, 400 µl of the high-titer virus was used to infect approximately 2×10^8^ cells. The cells were harvested three days after infection, and the cell pellets were stored at −20°C.

*Pb-Act2* and a 6×His-tag from the pETM-14 vector (EMBL) were amplified, and the inserts were assembled using multi-tier SLIC, as described before [28] into the pFastBac-Dual vector (Invitrogen), digested with *Eco*RI and *Xba*I. *Pb*-Act2 was produced in Sf21 (Invitrogen) cells essentially as described before [30]. Briefly, Sf21 cells were transfected with a bacmid generated in and isolated from the DH10MultiBac strain, using the FuGene 6 transfection reagent (Roche). The released virus particles were collected and used to generate a high-titer viral stock. Typically, 400 µl of the high-titer virus was used to infect 2×10^8^ cells. 60–70 h after the growth arrest, cells were harvested and stored at −20°C.

For purification of recombinant *Pf*-Act1 and *Pb*-Act2, the cells were resuspended in lysis buffer [20 mM *N*-cyclohexyl-2-aminoethanesulfonic acid pH 9.5, 250 mM NaCl, 1 mM adenosine-5′-triphosphate (ATP), 2 mM β-mercaptoethanol, 20 mM imidazole, 1× cOmplete Mini EDTA free (Roche)] and sonicated for 15 s. The supernatant was clarified by centrifugation at 18000 g and applied to Ni-NTA agarose (Qiagen). After extensive washing with the lysis buffer, the final washes were performed in a modified G-buffer [5 mM Tris-HCl (pH 8.0), 0.2 mM CaCl_2_, 0.5 mM ATP, and 2 mM β-mercaptoethanol], supplemented with 20 mM imidazole, and the bound proteins were eluted with the modified G-buffer, containing 300 mM imidazole. Subsequently, the proteins were dialyzed against G-buffer [5 mM Tris-HCl (pH 8.0), 0.2 mM CaCl_2_, 0.5 mM ATP, and 5 mM DTT] overnight at 4°C, concentrated, stored on ice, and used within a week.

### Skeletal muscle actin preparation

Pig loin muscle was obtained from pigs from the University of Oulu Laboratory Animal Centre. The study plan was reviewed and accepted by the local ethics committee of the University of Oulu Laboratory Animal Centre (decision number 096/11). According to the Finnish legislation, no official license for animal experiments was needed because the tissues were collected from animals euthanized using a method accepted by law. Because of ethical reasons, and to fulfil the principle of reduction, the tissues were collected from animals, which were used in other animal experiments, for which there was a separate license from the Finnish National Animal Experiment Board. Essentially, muscle acetone powder preparation and actin purification were performed as described previously [31]. Prior to use in experiments, SEC was carried out in G-buffer over a Superdex 200 16/60 column (GE Healthcare). Pure monomeric actin was stored on ice and used within a week.

### Synchrotron radiation circular dichroism spectroscopy

For synchrotron radiation circular dichroism (SRCD) measurements, the formin samples were dialyzed into 10 mM potassium phosphate buffer, pH 7.0, and the concentrations were adjusted to 1.1–1.5 mg/ml. SRCD data were measured on beamline CD1 at the ASTRID synchrotron storage ring, ISA, University of Aarhus, Denmark. Each sample was scanned from 280 to 170 nm in a 100-µm pathlength circular quartz cuvette. Three consecutive scans of each sample were performed, and the corresponding buffer spectrum was subtracted. SRCD data were processed using CDtool [32], and secondary structure deconvolution was carried out at the Dichroweb server [33], using the CDSSTR algorithm [34] and the SP175 reference database [35].

### Molecular weight determination by multi-angle static light scattering

Analytical SEC was carried out by injecting 200 µl of each of the three formin samples at 1 mg/ml into a Superdex 200 10/300 GL column (GE Healthcare) coupled to an Äkta Purifier (GE Healthcare). The running buffer was 50 mM sodium phosphate (pH 7.0), 50 mM NaCl, and 1 mM DTT, and the flow rate was 0.5 ml/min. The system was attached to a mini-DAWN TREOS multi-angle static light scattering (MALS) detector (Wyatt) and an Optilab rEX differential refractometer (Wyatt) for absolute molecular weight determination. Molecular weights were determined based on the measured light scattering and refractive index and/or UV absorbance using the ASTRA software (Wyatt).

### Small-angle scattering

Synchrotron small-angle X-ray scattering (SAXS) measurements were carried out on beamlines X33 (DORIS storage ring) and P12 (PETRA III) at EMBL/DESY, Hamburg, and on beamlines I711 and I911-4 at MAX-Lab, Lund. Protein concentrations in the measurements varied between 3–20 mg/ml, in a buffer containing either 20 mM Tris-HCl (pH 7.5), 150 mM NaCl, and 1 mM DTT (*Pf*-Frm1-FH1FH2) or 25 mM 4-(2-hydroxyethyl)-1-piperazineethanesulfonic acid (HEPES; pH 7.5), 150 mM NaCl, and 1 mM DTT (*Pf*-Frm1-FH2 and *Pf*-Frm1-FH2Δlasso). Programs of the ATSAS package [36] were used for data processing and analysis. 3D models were built using DAMMIN [37], DAMMIF [38], GASBOR [39], SASREF [40], and BUNCH [40]. SUPCOMB [41] and DAMAVER [42] were used for superpositions and the calculation of averaged models, respectively. A homology model of the *Pf*-Frm1 FH2 domain, consisting of 435 residues, including the core FH2 domain, the linker, and the lasso, was built based on PDB entry 3O4X [43] as a template, using the Phyre2 server [44]. The sequence alignment gives 23% sequence identity, and the Phyre confidence score for the model is 100%. For BUNCH modeling, the linker and lasso were removed from the homology model and rebuilt as a chain-like model. Rigid body modeling with SASREF was done using two approaches: First, the two protein chains from the homology model were used for rigid body refinement *per se*. Second, to mimic flexibility of the linker, while keeping the lasso-knob interaction in place, the dimer homology model was cut in half at each of the linker domains, between residues 104 and 105. Hence, rigid body refinement was carried out with two halves of the dimer, each of which contained, in addition to the FH2 domain, the lasso domain from the other subunit instead of the one from the same chain.

For small-angle neutron scattering (SANS), *Pf*-Frm1-FH1FH2 was dialyzed against 4 mM HEPES (pH 7.5), 20 mM NaCl, in 98% D_2_O. SANS data were collected on beamline SANS1 at GKSS (Geesthacht, Germany), and were processed on absolute scale.

### Biochemical assays

For co-sedimentation assays, 5 µM ATP-actin - either pig skeletal muscle actin or recombinant *Pf*-Act1/*Pb*-Act2 - was incubated in G- [5 mM Tris-HCl (pH 7.5), 0.2 mM CaCl_2_, 0.2 mM ATP, and 0.5 mM DTT] or F-buffer (G-buffer including 2 mM MgCl_2_, 50 mM KCl, and 2 mM ATP), alone and with 250 nM formins, at room temperature for 1–2 h. After ultracentrifugation at 435000 g for 60 min at 20°C, equal amounts of the supernatants and pellets were analyzed by sodium dodecyl sulphate polyacrylamide gel electrophoresis (SDS-PAGE) and Coomassie Brilliant Blue staining.

Actin polymerization was investigated at 22°C by fluorescence spectroscopy in a 200-µl quartz cuvette by measuring the change in fluorescence intensity (excitation 365 nm, emission 407 nm, bandpass 2 nm for both excitation and emission) with a Horiba Scientific FluoroMax-4 spectrofluorometer. Unlabeled pig muscle actin (5 µM) containing 5% pyrene-labeled rabbit actin (Cytoskeleton Inc.) was in G-buffer. Actin polymerization was initiated by adding 1/10 of the volume of 10× F-buffer (to a final concentration of 50 mM KCl, 2 mM MgCl_2_, and 2 mM ATP), in the presence of 0, 1, 5, 10, or 50 nM of the different *Pf*-Frm1 variants. The change in fluorescence intensity was followed for 1000 s at 10-s intervals. To assess the effect of *Pf*-Pfn on the polymerization kinetics, the assay was also performed with 5 and 10 µM *Pf*-Pfn in both presence and absence of 10 nM formins. The measurements were performed in duplicate, and averages of the individual measurements were compared.

## Results

### *Plasmodium falciparum* formin 1 forms stable dimers mediated by the lasso segment in solution

**Figure 1:**
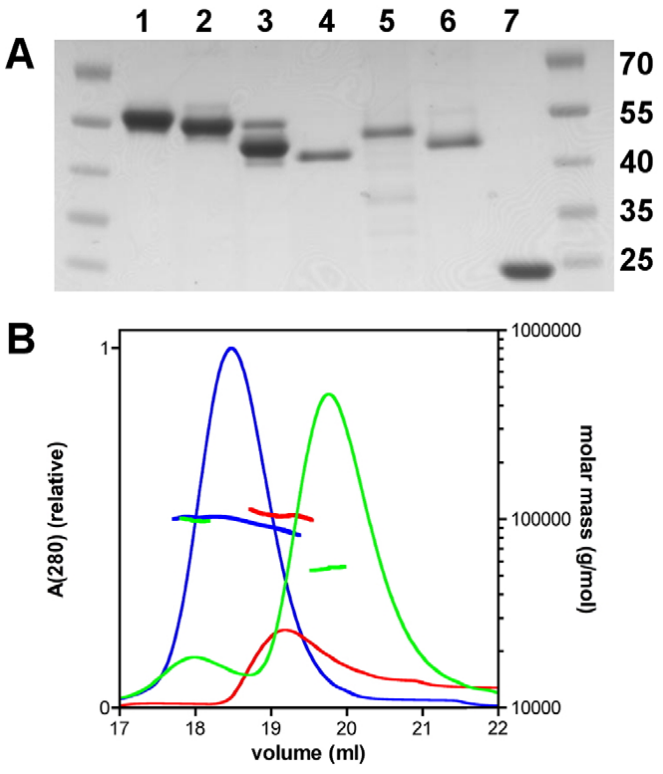
Purification and characterization of the recombinant proteins. **A**, SDS-PAGE analysis showing the purity of the proteins used in this study. The gel was stained using Coomassie Brilliant Blue. The samples are 1. *Pf*-Frm1-FH1FH2, 2. *Pf*-Frm1-FH2, 3. *Pf*-Frm1-FH2Δlasso, 4. pig muscle actin, 5. *Pf*-Act1, 6. *Pb*-Act2, and 7. *Pf*-Pfn. The molecular weights of the standard proteins in kDa are indicated on the right. The differences in the molecular weights of the *Plasmodium* and pig actins are accounted for by the 6×His-tags present in the recombinant *Plasmodium* actins. **B**, Size-exclusion chromatograms showing a single peak for *Pf*-Frm1-FH1FH2 (red) and *Pf*-Frm1-FH2 (blue) and two peaks for *Pf*-Frm1-FH2Δlasso (green). The molecular weights of the peaks of *Pf*-Frm1-FH1FH2 and *Pf*-Frm1-FH2 correspond to the size of dimers, and the two peaks of *Pf*-Frm1-FH2Δlasso to a dimer and a monomer, as determined by MALS (vertical lines with colors corresponding to those of the chromatograms).

We expressed and purified three different variants of *Pf*-Frm1 (Figure 1A): (i) the FH1-FH2 domains together (*Pf*-Frm1-FH1FH2), (ii) a longer version of the FH2 domain, which contains the so-called lasso region (*Pf*-Frm1-FH2), and (iii) a shorter version of the FH2 domain (*Pf*-Frm1-FH2Δlasso). SEC indicated an apparent dimer size for the two longer *Pf*-Frm1 proteins, whereas *Pf*-Frm1-FH2Δlasso appeared to be mainly monomeric, with a small fraction eluting earlier than the longer *Pf*-Frm1 dimers from the column (Figure 1B). However, as SEC does not give direct information on the molecular weight but rather the shape of the molecule, we used MALS to determine the true oligomeric state of each protein (Figure 1B, Table 1). The light scattering profiles, indeed, confirm that *Pf*-Frm1-FH1FH2 and *Pf*-Frm1-FH2 are dimeric in solution, with molecular weights of 105.0 kDa (±1%) and 98.2 kDa (±3%), respectively, whereas the majority of *Pf*-Frm1-FH2Δlasso is monomeric with a molecular weight of 54.9 kDa (±6%). The molecular weight of the small peak eluting earlier is 99.0 kDa (±6%), indicating that *Pf*-Frm1-FH2Δlasso also has the tendency to form dimers, which are not as tight and are less compact than the dimers of *Pf*-Frm1-FH1FH2 and *Pf*-Frm1-FH2.

**Table 1:** Properties of the three formin versions derived from SEC/MALS and SAXS.

Sample	Monomer MW* (kDa)	MW from SEC/MALS (kDa)	R_g_ **(nm)	D_max_ *** (nm)	Volume (nm^3^)	Chi, model *vs.* data (DAMMIN, DAMMIF, GASBOR, BUNCH)
*Pf*-Frm1-FH1FH2	57.7	105.0 (±1%)	6.4	25	352	0.9, 1.1, 1.1, 1.4
*Pf*-Frm1-FH2	52.3	98.2 (±3%)	5.2	19	335	1.2, 1.2, 1.7, -
*Pf*-Frm1-FH2Δlasso	48.8	major peak: 54.9 (±6%), minor peak: 99.0 (±6%)	6.7	23	299	1.3, 2.2, 2.1, -

The given volume is that of the respective averaged *ab initio* dummy bead model. The Chi-value describes the fit between the experimental scattering data and the model; with values close to unity reflecting a good fit.

* MW = molecular weight.

** R_g_ = radius of gyration.

*** D_max_ = maximum particle dimension.

**Figure 2:**
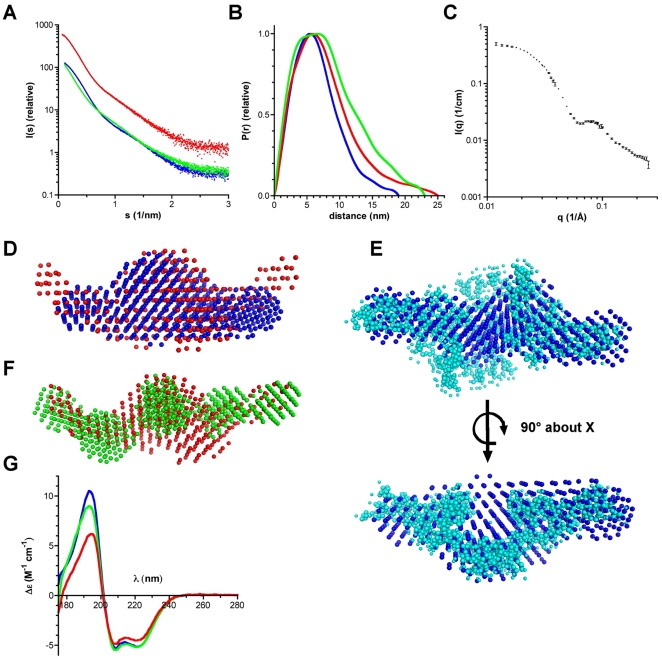
Solution structures of the *Pf*-Frm1 domains. **A**, Raw synchrotron SAXS data for *Pf*-Frm1-FH1FH2 (red), *Pf*-Frm1-FH2 (blue), and *Pf*-Frm1-FH2Δlasso (green). **B**, Distance distribution functions derived from the SAXS data; coloring as in panel A. **C**, SANS data for *Pf*-Frm1-FH1FH2. A radius of gyration (R_g_) of 5.7 nm and a maximum particle dimension (D_max_) of 18 nm can be estimated from the data. **D**, Averaged *ab initio* dummy atom models for *Pf*-Frm1-FH1FH2 (red) and *Pf*-Frm1-FH2 (blue) created by DAMMIN. The extensions at the extremities most likely correspond to the FH1 domain. **E**, Averaged *ab initio* models for *Pf*-Frm1-FH2 created by DAMMIN (blue) and GASBOR (cyan). The two figures are related by a 90° rotation about the X-axis. Both methods produce very similar models that fit the data. **F**, Averaged *ab initio* model for *Pf*-Frm1-FH2Δlasso (green) created by DAMMIN, superimposed on the structure of *Pf*-Frm1-FH1FH2 (red). Note that *Pf*-Frm1-FH2Δlasso is highly elongated, lacking a compact domain in the middle. **G**, SRCD spectra for the *Pf*-Frm1 variants. Coloring as in panel A. *Pf*-Frm1-FH2 has the highest relative helical content; see text for details on spectral deconvolution.

In order to confirm the above findings and to determine the 3D shapes of the different *Pf*-Frm1 variants, we studied their solution structures using synchrotron SAXS (Figure 2A,B). The basic data extracted from the SAXS scattering curves (Table 1) indicate that the construct with the lasso, but without the rudimentary FH1 domain (*Pf*-Frm1-FH2), is the most compact of the three constructs. While this was expected in comparison to *Pf*-Frm1-FH1FH2, it is surprising that the shorter construct, *Pf*-Frm1-FH2Δlasso, is more extended. The *Pf*-Frm1-FH1FH2 construct was also studied with SANS (Figure 2C). The neutron scattering curve had a similar shape to the SAXS curve, indicating the sample suffered no significant damage from X-rays during the SAXS measurement.

*Ab initio* model building further confirmed the presence of a compact dimer in the *Pf*-Frm1-FH2 construct. The longer *Pf*-Frm1-FH1FH2 is similar, with slightly longer dimensions, in line with the presence of 30 additional N-terminal amino acids, predicted to be unfolded (Figure 2D,E). However, the shortest construct, *Pf*-Frm1-FH2Δlasso, is significantly different, being less compact (Figure 2F). While it appeared to be largely dimeric at the concentration used in the SAXS experiments, no compact dimer, such as that seen in the other constructs, was observed. The *ab initio* model built based on the data is elongated, S-shaped, and thin. Furthermore, analysis of the SAXS data indicates this construct to most likely be present as a mixture of monomeric and dimeric forms, which is in line with the SEC/MALS results. Taken together, the data clearly show that the absence of the lasso segment interferes with the dimerization of the formin FH2 domain in solution, resulting also in loss of function of the protein, as presented below.

We also used SRCD spectroscopy to assess the folding state and secondary structure composition of the different formin variants (Figure 2G). *Pf*-Frm1-FH1FH2 contains 501 amino acid residues, and according to the deconvolution, out of these, 46% (230 residues) are in an α-helical conformation, 9% are in β strands, 13% in turns, and 31% (155 residues) do not form any secondary structure. As FH2 domains are mainly helical, this result is in agreement with known FH2 domain structures and secondary structure predictions. *Pf*-Frm1-FH2 consists of 450 residues, of which approximately 234 are in α helices and 113 are unordered. The result clearly proves that the FH1 domain is unfolded. *Pf*-Frm1-FH2Δlasso has 420 residues, of which approximately 223 are in α helices and 113 unordered. In addition to the predicted presence of a short helical stretch in the lasso, it is likely that the lack of the lasso segment and proper dimerization in the shortest construct leads to some degree of unfolding. This is also in line with the SAXS data, showing a more elongated structure than for the lasso-containing construct.

### *Plasmodium falciparum* formin 1 nucleates actin filaments, and the lasso-mediated dimerization is required for this activity

**Figure 3:**
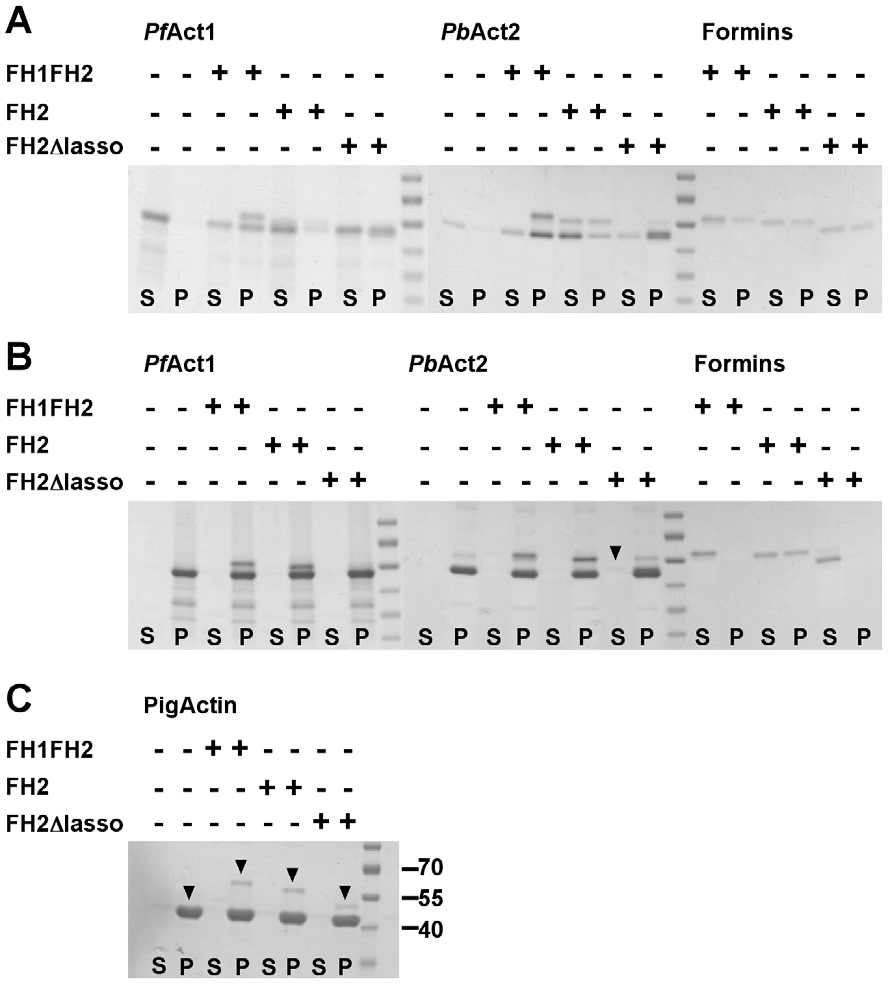
Co-sedimentation of *Pf*-Frm1 with *Plasmodium* and pig actins. **A**, *Pf*-Frm1 induces actin polymerization in low-salt conditions. *Pf*-Act1 and *Pb*-Act2 were incubated alone and in the presence of *Pf*-Frm1-FH1FH2, *Pf*-Frm1-FH2, and *Pf*-Frm1-FH2Δlasso in G-buffer, followed by ultracentrifugation at 435000 g. The supernatants and pellets were separated, and equal amounts were applied to the SDS gel, which was stained with Coomassie Brilliant Blue. The supernatants and pellets are labeled at the bottom of the gel as S and P, respectively. The presence or absence of the different *Pf*-Frm versions is indicated with + and − signs above. The first eight lanes contain *Pf*-Act1, the next eight *Pb*-Act2, and the last six only the formins in G-buffer. **B**, All three *Pf*-Frm versions associate with *Plasmodium* actin filaments. The actins were allowed to polymerize in the presence or absence of the formins in F-buffer and separated on SDS-PAGE, as in panel A. The first eight lanes contain *Pf*-Act1, the next eight *Pb*-Act2, and the last six only the formins in F-buffer. The arrowhead shows the small amount of *Pf*-Frm1-FH2Δlasso left in the soluble fraction in the presence of *Pb*-Act2 in F-buffer. **C**, All three formins also co-sediment with pig muscle actin. The experiment was performed as in panel B, and the presence or absence of the formins is indicated above the figure. The molecular weight standards of 70, 55, and 40 kDa are indicated. The same standards were used in all gels shown. The arrowheads indicate the positions of actin, *Pf*-Frm1-FH1FH2, *Pf*-Frm1-FH2, and *Pf*-Frm1-FH2Δlasso in the gel, from left to right.

It has previously been shown that the FH1-FH2 and FH2 domains of *Pf*-Frm1 nucleate filaments of chicken actin [6]. To evaluate the activities of the three *Pf*-Frm1 variants on *Plasmodium* actins, we performed co-sedimentation assays by ultracentrifugation, using recombinant *Pf*-Act1 and *Pb*-Act2. In low-salt conditions (G-buffer), we see that all three *Pf*-Frm1 versions induce the *Plasmodium* actins to polymerize to some extent (Figure 3A). All three formins appear to alone partially pellet in the low-salt condition, indicating that they are not very stable in the Tris-HCl buffer in the absence of salt. However, a significant part of the proteins is soluble also in G-buffer and upon addition of actin completely moves to the pellet fraction, taking along approximately half of *Pf*-Act1 and *Pb*-Act2, which both alone in G-buffer are completely soluble.

In typical polymerization conditions with a high salt concentration (F-buffer), both *Plasmodium* actins polymerize completely under the conditions used in the assay. All three *Pf*-Frm1 versions nearly quantitatively co-sediment with the actin filaments (Figure 3B), while they are mostly soluble in F-buffer in the absence of actin, proving that they tightly associate with actin filaments. There is some *Pf*-Frm1-FH2Δlasso left in the soluble fraction in F-buffer with actin. However, it is not possible to see how much of it is in the pellet, as *Pf*-Frm1-FH2Δlasso is not well separated from the *Plasmodium* actins on the gels. As a control, the experiment in F-buffer was also performed using pig skeletal muscle actin. As expected, all formins associate with the actin filaments and are found in the pellet fractions (Figure 3C). It is noteworthy that the pig actin allows for a better separation of the actin and *Pf*-Frm1-FH2Δlasso.

**Figure 4:**
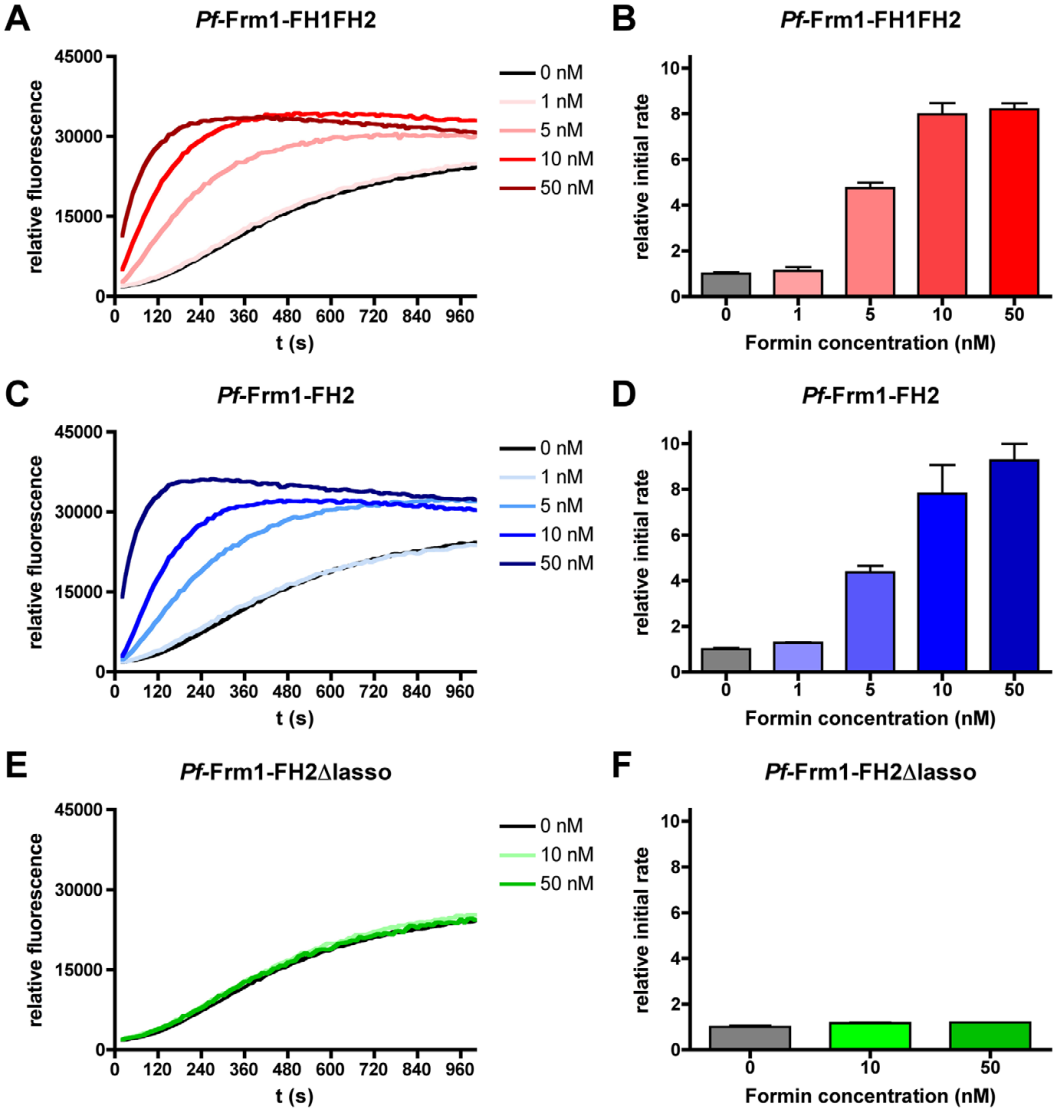
Effect of *Pf*-Frm1 on actin polymerization kinetics. The effect of *Pf*-Frm1 on the kinetics of actin polymerization was tested by measuring the change in fluorescence upon incorporation of 5% pyrene-actin into growing actin polymers. The actin concentration used in all experiments was 5 µM. The initial rates (ΔF/s) were calculated as the slope of the linear part (120 s) of the fluorescence curves. In order to facilitate comparison, the samples containing only actin were set to the value 1. **A–B**, Different concentrations of *Pf*-Frm1-FH1FH2. **C–D**, Different concentrations of *Pf*-Frm1-FH2. **E–F**, Different concentrations of *Pf*-Frm1-FH2Δlasso.

To further characterize the activities of the different length formins, we performed polymerization assays using pig muscle actin and monitoring the increase in fluorescence upon co-polymerization of 5% pyrene-labeled actin (Figure 4). Already 5 nM *Pf*-Frm1-FH1FH2 and *Pf*-Frm1-FH2 significantly accelerated the initial rate of polymerization (Figures 4A–D), indicating participation in the nucleation of actin filaments. However, *Pf*-Frm1-FH2Δlasso had no significant effect on the polymerization rate at the concentrations used (Figure 4E,F). Thus, the FH2 domain dimer, mediated by the lasso region, is both necessary and sufficient for the nucleation activity of *Pf*-Frm1.

### *Plasmodium falciparum* profilin sequesters actin monomers alone but has little effect on actin polymerization in the presence of formin 1

**Figure 5:**
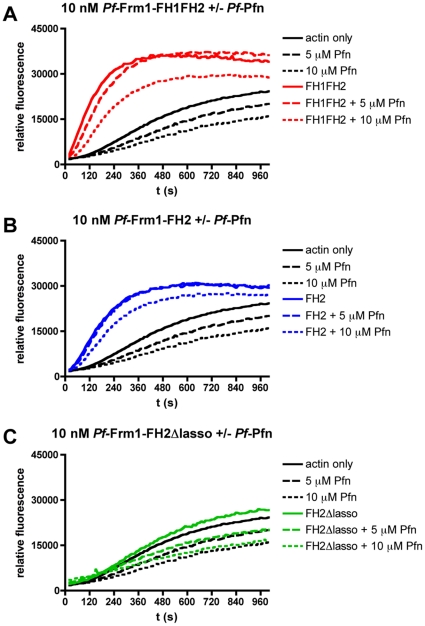
Effect of *Pf*-Pfn on actin polymerization kinetics in the presence of *Pf*-Frm1. The effect of *Pf*-Pfn added at a 1∶1 or 2∶1 molar ratio to actin on the kinetics of actin polymerization in the presence of the different *Pf*-Frm1 domains was tested by measuring the change in fluorescence upon incorporation of 5% pyrene-actin into growing actin polymers. The actin concentration used in all experiments was 5 µM. **A**, *Pf*-Pfn together with 10 nM *Pf*-Frm1-FH1FH2. **B**, *Pf*-Pfn together with 10 nM *Pf*-Frm1-FH2. **C**, *Pf*-Pfn together with 10 nM *Pf*-Frm1-FH2Δlasso.

In order to assess whether *Plasmodium* profilin works together with *Pf*-Frm1 to accelerate actin polymerization, we performed pyrene-actin fluorescence polymerization assays also in the presence of *Pf*-Pfn. As expected, based on the function of profilins from other species, *Pf*-Pfn alone inhibits actin polymerization (Figure 5). However, together with the different *Pf*-Frm1 domains used, *Pf*-Pfn at an equimolar ratio to actin had little or no effect either on the initial rate or the total amount of actin polymerization (Figure 5). At a 2∶1 profilin-actin ratio, profilin seems to slightly inhibit actin polymerization also in the presence of formins. Thus, we can conclude that *Pf*-Pfn either binds to actin with a lower affinity than *Pf*-Frm1 or that *Pf*-Frm1 nucleates and elongates profilin-actin even in the absence of a canonical profilin-binding FH1 domain.

## Discussion

Our current view of the actin-based motility of apicomplexan parasites is somewhat controversial; despite the importance of rapid formation of actin filaments, most of apicomplexan actin is present in the monomeric state [5]. The system also seems rather vulnerable due to the very small number of actin-binding proteins taking part in the rapid treadmilling of actin in the parasites. Especially perplexing is the lack of most of the conventional actin nucleators - while fast nucleation would seem more important than elongation in a system, where only very short, transient actin filaments are required. In essence, the only candidates for actin nucleation in *Plasmodium* and other *Apicomplexa* are the formins. Formins in most organisms work as nucleators and processive cappers, protecting the barbed end from capping proteins and accelerating the rate of polymerization by recruiting profilin-actin complexes *via* their proline-rich FH1 domains [19]. We show here that *Pf*-Frm1 promotes actin filament assembly on both mammalian skeletal muscle and recombinant *Plasmodium* actins, suggesting a major role in actin filament nucleation in *Plasmodium*.

Apicomplexan actins have been reported to polymerize weakly compared to conventional actins [5], [45]. Yet, we see both *Plasmodium* actins almost completely polymerizing in the pelleting assay, in the absence of any nucleators or filament-stabilizing agents. All three *Pf*-Frm1 constructs studied here associate with the actin filaments and, interestingly, nearly completely co-sediment with the filaments, indicating that, in addition to the growing ends, they may also bind to the sides of the filaments, as has been suggested earlier for *e.g.* the *Drosophila melanogaster* formin DAAM [46]. Although bundling has not been shown to happen in apicomplexan actins, this raises the question whether *Pf*-Frm1 could play a role in bundling and cross-linking of the short parasite actin filaments.

We show that dimerization of the *Pf*-Frm1 FH2 domain is mediated by the lasso region N terminal to the core FH2 domain, and the formation of a compact FH2 dimer is important for *Pf*-Frm1 activity. Furthermore, the *Pf*-Frm1 FH2 domain also weakly induces actin polymerization in low-salt conditions, which indicates a strong interaction and nucleating activity with actin. In our assays, approximately 5 nM *Pf*-Frm1-FH1FH2 and *Pf*-Frm1-FH2 were sufficient for an increase in the initial actin polymerization rate. This is in agreement with what has been reported before for both *Plasmodium* and *Toxoplasma* formins, where low nanomolar formin concentrations were enough for a significant increase in the polymerization rates [6], [27].

*Pf*-Frm1 lacks a well-defined FH1 domain and has, instead, only few proline residues scattered in the region preceding the FH2 domain. Consistent with the lack of proline-rich repeats in the *Pf*-Frm1 sequence, we have not detected an interaction between *Pf*-Frm1 and *Pf*-Pfn, using purified recombinant proteins in GST pull-down assays and SEC (unpublished data). Therefore, it is not unexpected that *Pf*-Pfn had very little, if any, effect on actin polymerization kinetics in the presence of *Pf*-Frm1. It seems likely that the role of *Pf*-Pfn is, indeed, to efficiently bind actin monomers in a form that facilitates their incorporation to the barbed ends of the growing actin filaments.

Previously, we have shown that *Pf*-Pfn binds to proline-rich sequences [7]. Interestingly, the *Plasmodium* formin 2 isoform contains in its putative FH1 domain two potential profilin-binding regions. It is remarkable that, although *T. gondii* profilin is highly conserved with *Pf*-Pfn, it has been reported to be unable to bind proline-rich sequences [27], [47]. The apicomplexan profilins are very distantly related to profilins from other *phyla* but the *P. falciparum* and *T. gondii* profilins are 42% identical. In *Pf*-Pfn, the aromatic nature of the proline-rich-peptide binding site is conserved but many of the exact amino acid positions are not [7]. The same is true for *T. gondii* profilin [47], and furthermore, there are differences in the aromatic residues of the peptide-binding surface of these two apicomplexan profilins, as well. Therefore, it is possible that they have somewhat divergent functions and mechanisms. Despite the similarities in the actin structures and their regulation in these two parasites, there are also significant differences, which at least partly might be due to the very different life cycles of *Plasmodium* and *Toxoplasma*. Whereas *Toxoplasma* only uses mammalian hosts and displays virtually no cell specificity, *Plasmodium* needs both a mammalian and an arthropod host and is highly specific with regard to its host cells, depending on the life cycle stage. Furthermore, *Plasmodium* has two actin isoforms and *Toxoplasma* only one [48]–[50]. Therefore, the differences in the actin regulatory proteins may reflect adaptations to both differential actin isoforms and also different mechanisms needed to enter and survive within different host cells.

Our data support the role of a G-actin sequesterer for *Plasmodium* profilin but do not exclude the possibility that it works together with *Plasmodium* formin 2 to accelerate elongation, like in most other organisms. This implies that *Plasmodium* could have two diverged formins; one functioning rather as a sole nucleator and the other one also as an elongation factor together with profilin. Apparently, in *Toxoplasma*, such a difference does not exist [27]. For *Plasmodium*, this remains to be seen, once *Plasmodium* formin 2 has been properly characterized.

**Figure 6:**
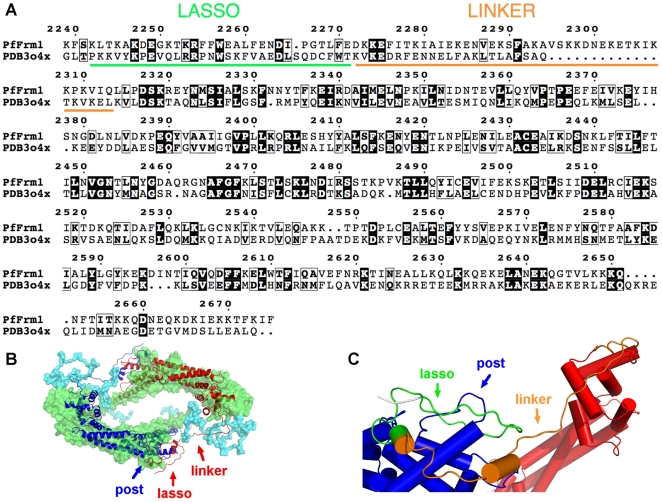
Model for the dimerization of *Plasmodium* formin 1. **A**, Sequence alignment between the *Pf*-Frm1 FH2 domain and the best template identified by Phyre for homology modeling (mouse mDia1 [43]). *Pf*-Frm1-FH2 includes both the lasso and linker domains, while *Pf*-Frm1-FH2Δlasso has only the linker segment. **B**, Comparison of the dimer homology model of *Pf*-Frm1 (shown as cartoons; the dimerization in the model is built identical to that seen in the mDia1 template structure 3O4X [43]) with the SAXS model built by using the core FH2 domains as rigid bodies (green) and building the linker, lasso, and FH1 segments by chain-like assemblies of dummy residues (cyan). The SAXS model was made with BUNCH, applying P2 symmetry, but no distance restraints. The Chi-value against the raw SAXS data is 1.4, reflecting a very good fit to the measurement; the fit is shown in Figure S1B. Figure S1 also contains further rigid body refinement analysis of the homology model. **C**, A close-up view into the model of the lasso segment (green) in a *Pf*-Frm1 dimer; the FH2 domain monomers are indicated in blue and red. The orange segment represents the flexible linker region.

From the structural point of view, our data indicate that the dimeric structure of *Pf*-Frm1-FH2 in solution is similar to that seen *e.g.* in the crystal structure of the mouse mDia1 (PDB code 3O4X) [43] (Figure 6). The mouse mDia1 and *Pf*-Frm1 FH2 domains have 23% sequence identity (Figure 6A). The flexible linker region between the lasso segment and the core FH2 domain provides adaptability that is required for interactions with actin; in the actin-bound state, two actin monomers are bound between the core FH2 domains. Modeling of *Pf*-Frm1-FH1FH2 using the core FH2 domain and chain-like fragments, with the BUNCH software, provides also a dimeric model, which fits the scattering data very well, while preserving the expected intermolecular interactions (Figure 6B). From other formin FH2 domain structures, it is predicted that the lasso segment wraps around the so-called post region of the opposing FH2 monomer [24], [43], [51]–[53] (Figure 6C). From rigid body modeling using a homology model of the dimer (Figure S1), it is seen that rather minor rearrangements of the mDia1 dimer, which can be obtained from some flexibility in the linker region, result in a model that fits the SAXS data very well. The head-to-tail 2-fold symmetrical arrangement of the FH2 domain dimer, combined with the expected flexibility of the linker region between the FH2 and the lasso domains, provides a versatile framework for the binding of actin dimers by the dimerized FH2 domain. In the co-sedimentation assays (Figure 3) we see that all three formin versions associate with actin filaments and also induce formation of filaments in low-salt conditions. However, in the pyrene-actin fluorescence polymerization assay (Figure 4), only the two longer, dimeric versions increase the polymerization rate. It is possible that the FH2 domain in the absence of the lasso and, thus, proper dimerization still binds to actin filaments, as seen before for mouse mDia1 and mDia3 core FH2 domains [23]. Our results indicate that *Pf*-Frm1-FH2Δlasso is also somehow capable of bringing together actin monomers as short filament nuclei or aggregates, which are large enough to sediment at 435000 g, but cannot support further elongation of the filaments.

Our SAXS and SRCD data also prove that, as predicted based on the sequence, but to our knowledge not visualized for any formin before, the FH1-like domain of *Pf*-Frm1 is unstructured, and presents itself as two protrusions at opposite ends of the head-to-tail FH2 domain dimer.

### Concluding remarks

We have shown that *Pf*-Frm1 is capable of initiating actin filament formation on both skeletal muscle as well as *Plasmodium* actins. This is the first time that such a polymerization promoting effect has been shown for purified, recombinant *Plasmodium* actins. This confirms *Pf*-Frm1 to be the prime candidate responsible for actin filament nucleation in these parasites. Our data also show that, while *Pf*-Pfn efficiently sequesters monomers, its monomer sequestering activity does not compete with the nucleation/elongation activities of *Pf*-Frm1. This, indeed, emphasizes the role of profilin in providing polymerization competent actin monomers to be added to the growing end of an actin filament – probably even in the absence of a direct interaction between profilin and a formin FH1 domain.

In addition, we provide the first structural insight into the dimerization of an apicomplexan formin and prove its relevance for the actin polymerizing activity. The solution structures presented here serve as a first step towards understanding the structure-function relationships in these multidomain proteins. In order to understand the complex interplay between the malaria parasite formins, actin, and other regulatory proteins interacting with them, more structural work at different levels of resolution is of crucial importance.

## Supporting Information

Figure S1 **Comparison of different models of** ***Pf*** **-Frm1 FH2 domain to the SAXS data.** **A**, Three models are shown: orange, the homology model based on the mDia1 structure (PDB entry 3O4X [43]) – also shown in Fig. 6B superimposed on the BUNCH model; green, a model of the dimer obtained by rigid body refinement of the two FH2 domain chains from the homology model; blue, a rigid body model obtained from two halves of the dimer, such that there is a cut in the linker between residues 104 and 105 (blue arrows). Hence, in the refinement, the lasso belongs to the same rigid body as the corresponding knob. The locations of the lasso segments are indicated by circles. The two monomers in each model are colored slightly differently for clarity. **B**, Fit (red) of the BUNCH model shown in Fig. 6B to the scattering data of *Pf*-Frm1-FH1FH2 (black dots). **C**, Fits of the 3 models shown in A to the scattering data of *Pf*-Frm1-FH2, corresponding to the model. Coloring of the fits corresponds to the coloring of the respective models in A. The small changes in the dimer conformation in the rigid body models improve the fit significantly.
(TIF)
