# Supplementary figures and images for: The Lasso Segment Is Required for Functional Dimerization of the Plasmodium Formin 1 FH2 Domain
Source: PLoS One. 2012 Mar 13;7(3):e33586. doi: 10.1371/journal.pone.0033586 (PMC3302767; doi:10.1371/journal.pone.0033586)

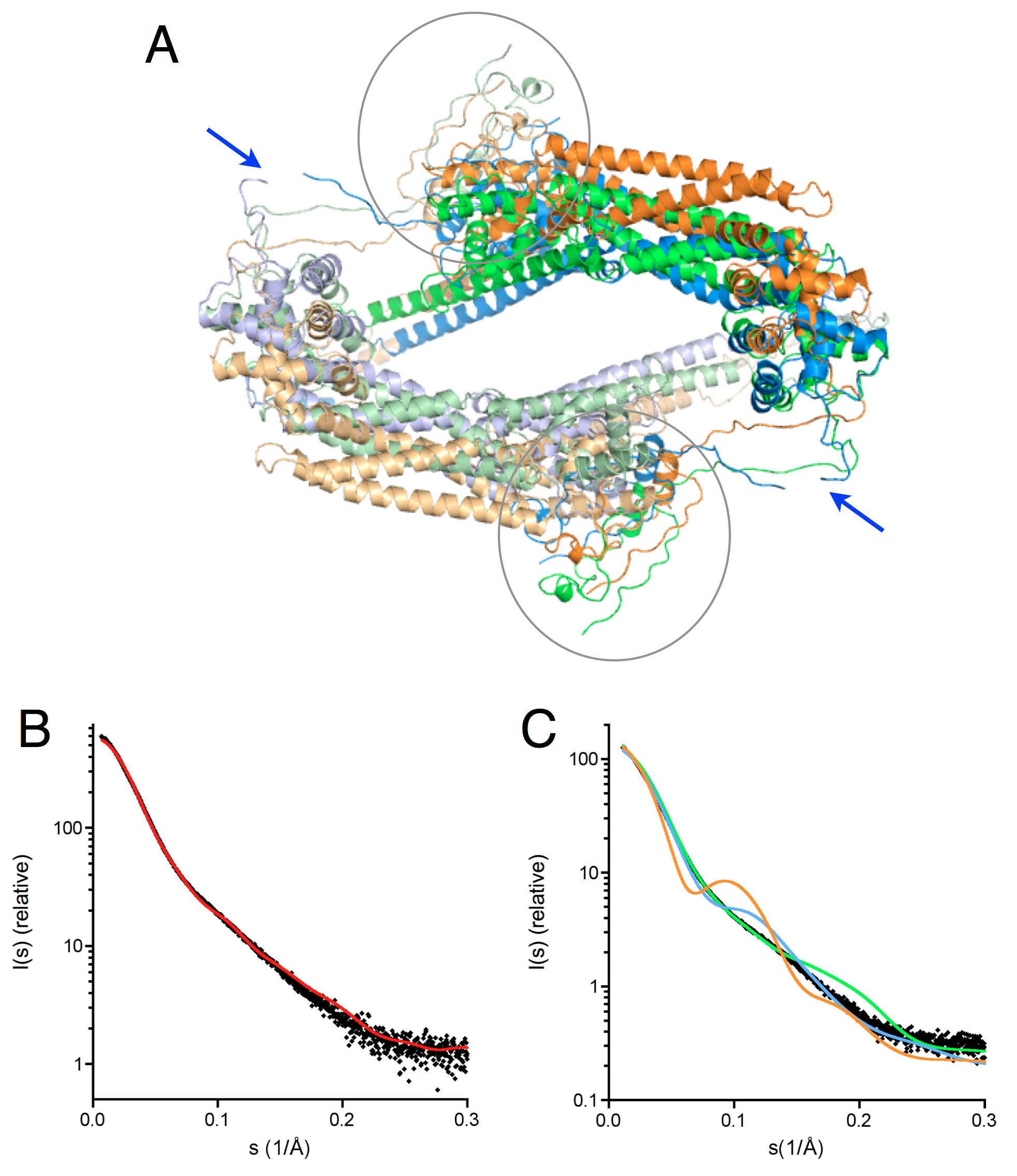

Supplement: Figure S1 — Comparison of different models of Pf -Frm1 FH2 domain to the SAXS data. A, Three models are shown: orange, the homology model based on the mDia1 structure (PDB entry 3O4X [43]) – also shown in Fig. 6B superimposed on the BUNCH model; green, a model of the dimer obtained by rigid body refinement of the two FH2 domain chains from the homology model; blue, a rigid body model obtained from two halves of the dimer, such that there is a cut in the linker between residues 104 and 105 (blue arrows). Hence, in the refinement, the lasso belongs to the same rigid body as the corresponding knob. The locations of the lasso segments are indicated by circles. The two monomers in each model are colored slightly differently for clarity. B, Fit (red) of the BUNCH model shown in Fig. 6B to the scattering data of Pf-Frm1-FH1FH2 (black dots). C, Fits of the 3 models shown in A to the scattering data of Pf-Frm1-FH2, corresponding to the model. Coloring of the fits corresponds to the coloring of the respective models in A. The small changes in the dimer conformation in the rigid body models improve the fit significantly. (TIF) [file pone.0033586.s001.tif]
